# Circular RNAs: Non-Canonical Observations on Non-Canonical RNAs

**DOI:** 10.3390/cells12020323

**Published:** 2023-01-14

**Authors:** Brett W. Stringer, Laura Gantley, Simon J. Conn

**Affiliations:** Flinders Health and Medical Research Institute (FHMRI), College of Medicine and Public Health, Flinders University, Adelaide, SA 5042, Australia

**Keywords:** circular RNA, circRNA, back splicing, biomarkers, transcriptomics, RNA regulation

## Abstract

The existence of circular RNA (circRNA) research in mainstream science can be attributed to the contemporary synergism of big data and keen attention to detail by several research groups worldwide. Since the re-emergence of these non-canonical RNA transcripts, seminal advances have been made in understanding their biogenesis, interactome, and functions in diverse fields and a myriad of human diseases. However, most research outputs to date have focused on the ability of highly stable circRNAs to interact with, and impact signalling through, microRNAs. This is likely to be the result of seminal papers in the field ascribing a few remarkable circRNAs as “miRNA sponges”. However, the stoichiometric ratio between the (often-lowly-expressed) circRNA and their (commonly-more-abundant) target is rarely in favour of a biologically relevant and functional consequence of these interactions. It is time for yet another revolution in circRNA research to uncover functions beyond their documented ability to bind miRNAs. This Special Issue aims to highlight non-canonical functions for this non-canonical family of RNA molecules.

## 1. Introduction

Circular RNA (circRNA), as the name suggests, is RNA that exists in a covalently- closed state rather than the more familiar, or canonical, linear form [1]. This property enhances its stability and increases its half-life compared to linear RNA [2]. Yet despite this, circRNA is a rare form of RNA, accounting for just 0.1% of total RNA within eukaryotic cells [3]. circRNAs were first visualised by electron microscopy in the 1970s [4] and then documented by Sanger sequencing in the 1990s, with them being referred to simply as scrambled exons [5]. Interest in circRNAs has recently experienced something of a renaissance, fuelled by the growth of RNA-sequencing and the emergence of tools to interrogate this abundant data. The vast majority of circRNAs are formed by a process known as back-splicing, which is the splicing of a downstream exon to an upstream one, thus forming a closed circle of single stranded RNA. CircRNAs formed in this manner are a theoretical possibility for any gene with one or more introns, yet not all transcribed, spliced genes produce circRNAs [6]. Indeed, the expression of circRNA is dynamic and tightly regulated—including species conservation and expression independent of parental mRNA [7,8]. This ancient and conserved gene expression phenomena implies functions for circRNAs. For this reason, the collection of articles in this Special Issue is particularly important, with the articles shedding light on the expanding repertoire of circRNAs and their formation, deep transcriptomic characterisation, and function.

This Special Issue entitled “Circular RNAs: Non-Canonical Observations on Non-Canonical RNAs” comprises 14 articles that are briefly summarised and contextualised below (Table 1).

## 2. Cardiovascular circRNAs

Cardiovascular disease remains the major cause of death and the cause of considerable morbidity in many countries around the world and circRNAs found in serum are potential biomarkers of such disease. Wang & Liu [12] provide a detailed review of circRNAs and their functions in diseased heart, with their review focusing on heart failure and arrhythmias. As a completely novel resource, Jakobi et al. [17] created a cardiovascular circRNA dataset from human-induced pluripotential stem cells differentiated towards cardiac myocytes, human umbilical vein endothelial cells, and healthy human tissue and diseased heart tissue. Deep sequencing of ribosomal RNA-depleted RNA enriched for circRNA was carried out by RNase A treatment. Analysis of this resource identified shared and model-specific circRNA expression. Sixty-three of the identified circRNAs were found to be expressed in both pig and mouse hearts. Significantly, sequencing revealed fewer exons within circRNAs than expected from the genomic structure. This evidence of alternative splicing within circRNAs has important implications for investigating circRNA function. Cardiac datasets of Ago2 binding sites mapped to <7% of circRNAs showed little evidence of miRNA binding by association with the RISC complex, while ribosome binding sites mapped to <1% of circRNAs showed even less evidence of translation. Nevertheless, a novel class of circRNAs in this dataset contained the start codon of the host gene within the body of the circRNA. These AUG circRNAs were expressed at higher levels than non-AUG circRNAs. Intriguingly, ribosome profiling showed reduced translation of AUG circRNA host genes compared with non-AUG circRNA host genes, with the focus shifting from translated circRNAs to an effect induced by circRNAs on host gene translation.

## 3. Neuronal circRNAs

Alternative splicing is a feature of eukaryotic transcription, particularly within the nervous system. Gasparini et al. [9] reviewed major aspects of circRNA formation, metabolism, and function, and they proposed new functions in the context of the nervous system. Within neuronal cells, RNA splicing frequently introduces microexons, 3–30 nucleotide sequences that are not part of the canonical gene exon structure, into processed linear mRNA transcripts, thus expanding the functional potential of the encoded protein. Conn et al. [18] showed for the first time that microexon (ME) inclusion also occurs in circRNA, including at back-splice junctions. While ME-circRNAs can be readily detected using common circRNA prediction pipelines such as CIRCexplorer2 and CIRI2, a bespoke pipeline referred to as Hyb was used to identify microexon inclusion at back-splice junctions. By creating a circRNA dataset from ribosome-depleted, RNase R-digested RNA from gliomas of varying grades, the researchers found that circRNAs have prognostic potential as biomarkers to stratify gliomas from normal brain tissue. They also demonstrated that expression of the splicing-associated factor serine/arginine repetitive matrix 4 (SRRM4), known to regulate the inclusion of microexons in mRNA, was correlated with ME-circRNA abundance in gliomas, and when overexpressed in HEK293 cells, it resulted in the genesis of over 2000 novel ME-circRNAs. Given that microexons and circRNAs have been implicated in oncogenesis, the identification of ME-circRNAs adds to the functional potential of these enigmatic RNAs.

## 4. Non-Canonical circRNAs

Not all circRNAs are produced by back-splicing (exonic circRNAs). Robic et al. [19] investigated non-canonical circRNAs in a dataset from porcine testis, an abundant source of long non-coding RNA (lncRNA), that are also known to contain a large number of circRNAs including intron-derived circRNAs. There are challenges associated with studying non-canonical circRNAs using alignment tools best adapted to the analysis of exonic circRNAs and less comprehensively annotated lncRNAs; nevertheless, the team discovered that non-canonical circRNAs arose from two sources: intronic sequences and mono-exonic genes, with the latter being primarily non-coding. Interestingly, some non-canonical circRNAs, termed by the authors as sub-exonic circRNAs, contained partial exon sequences. Few canonical circRNAs were produced from long non-coding RNAs. The authors conclude that most non-canonical circRNAs arise from non-coding RNAs.

## 5. circRNAs as Disease Biomarkers

Due to their lack of 5′ or 3′ termini, circRNAs are known to be more stable, manifesting as a longer half-life, compared to their linear counterparts. Combining this with the fact that many circRNAs are tissue-specific and disease-specific, they present as strong candidates as biomarkers of disease—both prognostically and diagnostically. This exciting area, including the formation, clinical relevance, and therapeutic potential for circRNA biomarkers, has been reviewed for diabetes mellitus and related cardiovascular complications by Zaoui [11]; for sepsis by Beltrán-García et al. [13]; for schizophrenia by Nedoluzhko et al. [14]; and, as previously mentioned, for cardiovascular disease by Wang and Liu [12].

## 6. circRNAs in Oral Cancer

Moving beyond circRNAs as biomarkers, Chen et al. [20] identified a functional pro-tumourigenic circRNA in oral squamous cell carcinoma (OSCC) and linked its phenotypic effects with the regulated transcription of tumour-associated genes. Performing RNA-seq of 39 matched pairs of tumours and adjacent normal tissue, the researchers identified almost 114,000 circRNAs; 443 of these were found to be differentially expressed in tumours compared with normal tissues (207 upregulated and 236 downregulated, derived from 382 parental coding genes). Focusing on previously uncharacterised circRNAs, the researchers validated the upregulation of circFLNB 2-4 in OSCC versus the adjacent normal tissue by RT-PCR. Using two OSCC cell lines, they went on to show that back-splice-targeted, shRNA-mediated knockdown of circFLNB 2-4 reduced tumour cell proliferation, colony formation, cell migration and invasion, and subcutaneous tumour formation and increased sensitivity to doxorubicin while circFLNB 2-4 overexpression improved tumour cell survival following exposure to doxorubicin, which is consistent with the pro-tumourigenic function of circFLNB 2-4 and a protective role against cytotoxic stress. Expression of the parental gene remained unchanged in both experimental conditions, thus suggesting that circFLNB 2-4 function was independent of FLNB. RNA-seq analysis of knockdown and control cells revealed significant downregulation of general oncogenic pathways in circFLNB 2-4 knockdown cells. Furthermore, intersection of this gene set with circFLNB co-expressed genes in the original patient dataset identified a set of 27 co-regulated genes linked to the phenotypic effects of circFLNB, whose expression is potentially regulated by circFLNB in promoting OSCC progression.

## 7. Protein-Related Roles of circRNAs

Wawrzyniak et al. [16] reviewed the protein-related roles of circRNAs in human pathologies. circRNAs not only regulate protein synthesis, destination, and degradation, but they can also serve as protein scaffolds or recruiters and produce short peptides, through IRES or N6-methyladenosine-(m6A)-mediated translation, with active biological functions. One such example was described recently by Mo et al. [21] who reported a circRNA encoding an amyloid beta peptide in Alzheimer’s disease (AD). CircAβ-a harbours the Aβ-coding region of the APP gene. It was detected in the brains of AD patients as well as in non-dementia controls. Mo et al. [21] demonstrated that it is efficiently translated into a novel Aβ-containing Aβ175 polypeptide (19.2 kDa) in both cultured cells and human brain and that it is processed into Aβ peptides, a hallmark of AD. Interestingly, the circAβ-a-encoded polypeptide variant arises from APP coding exons 14–17 and an exon-14-derived C-terminus translated out-of-frame which the canonical mRNA could not have generated. The resultant unique 17-amino-acid C-terminal peptide fragment was detected by the researchers in human brain samples. This alternative pathway of Aβ biogenesis highlights circRNA as a novel evolutionary mechanism for increasing proteome complexity. This article was selected as the Editor’s choice.

## 8. Viral circRNAs

The first reported circRNAs were the family of small infectious genomes of viroids and satellites of plant viruses, some of which contain small self-cleaving RNA motifs, such as the hammerhead (HHR) and hairpin ribozymes. It has emerged that animals have been reported to contain hepatitis-delta-virus-(HDV)-like circRNAs with typical HDV ribozymes, but with conserved HHR motifs as well. De la Pena et al. [15] identified a second natural pathway of circRNA expression conserved in numerous plant and animal genomes, which efficiently promotes the accumulation of small non-coding RNA circles through the participation of HHRs. Most of these belong to a novel family of non-autonomous retrotransposons called retrozymes and in their review, the authors discuss their potential in a novel pathway of gene regulation.

Many viruses, including severe acute respiratory syndrome coronavirus 2 (SARS-CoV-2 or COVID-19) are single-stranded RNA viruses. De Falco et al. [10] summarised the extent of understanding around the circularisation of flavivirus genomes, focusing on dengue and Zika viruses. The authors focused on regions of the virus genomes and provided both structural and interactomic (RNA–RNA and RNA–protein) descriptions of the process required for the life cycle of the virus. The dependency of viral replication on this circularisation offers a novel target for therapies towards flaviviruses and other single-stranded RNA viruses.

## 9. circRNAs in Plants

While circRNAs are present across the eukaryotic tree of life, comparatively little is known about their role in plants. Philips et al. previously reported that most circRNAs in *Arabidopsis thaliana* are generated stochastically, and thus, they probably have no biological function [23]. As a part of their article featured this Special Issue, they investigated circRNA expression in 18 *A. thaliana* mutants of genes involved in splicing [22]. Focusing on circRNAs only produced in all experimental biological replicates, they identified three mutants that result in the accumulation of circRNAs. Two of these mutants also result in an expanded repertoire of circRNAs relative to a wild-type reference strain. Their findings suggest that one function of the spliceosome machinery is to prevent the stochastic generation of circRNA.

## 10. Conclusions

British evolutionary biologist and author Richard Dawkins recounts a story where a teacher at Oxford University told a junior colleague within the Science section of the Bodleian library, “Ah, dear boy, I see you are consulting the literature. Don’t. It will only confuse you” [24]. These words of caution might well be apt advice for someone about to wade into the circRNA literature. Indeed, a similar sentiment helped to motivate this *Cells* Special Issue. It is our hope, therefore, that the assembled collection of original and review articles will help to redress the sometimes-narrow view of the potential functions of circRNA and we hope that it will both enlighten and broaden readers’ understanding of this enigmatic class of RNA.

## Figures and Tables

**Table 1:** Articles published in this Special Issue entitled “Circular RNAs: Non-Canonical Observations on Non-Canonical RNAs”.

Author	Title	ArticleType	Ref.
Gasparini	The Secret Garden of Neuronal circRNAs	Review	[9]
De Falco	The Pseudo-Circular Genomes of Flaviviruses: Structures, Mechanisms, and Functions of Circularization	Review	[10]
Zaiou	circRNAs Signature as Potential Diagnostic and Prognostic Biomarker for Diabetes Mellitus and Related Cardiovascular Complications	Review	[11]
Wang and Liu	Circular RNA in Diseased Heart	Review	[12]
Beltrán-García	Circular RNAs in Sepsis: Biogenesis, Function, and Clinical Significance	Review	[13]
Nedoluzhko	The Biomarker and Therapeutic Potential of Circular RNAs in Schizophrenia	Review	[14]
de la Peña	A Singular and Widespread Group of Mobile Genetic Elements: RNA Circles with Autocatalytic Ribozymes	Review	[15]
Wawrzyniak	Protein-Related Circular RNAs in Human Pathologies	Review	[16]
Jakobi	Deep Characterization of Circular RNAs from Human Cardiovascular Cell Models and Cardiac Tissue	Article	[17]
Conn	SRRM4 Expands the Repertoire of Circular RNAs by Regulating Microexon Inclusion	Article	[18]
Robic	In-Depth Analysis Reveals Production of Circular RNAs from Non-Coding Sequences	Article	[19]
Chen	circRNAome Profiling in Oral Carcinoma Unveils a Novel circFLNB that Mediates Tumour Growth-Regulating Transcriptional Response	Article	[20]
Mo ^1^	Circular RNA Encoded Amyloid Beta peptides-A Novel Putative Player in Alzheimer’s Disease	Article	[21]
Philips	*Arabidopsis thaliana* cbp80, c2h2, and flk Knockout Mutants Accumulate Increased Amounts of Circular RNAs	Article	[22]

^1^ Editor’s choice.

## References

[1] Kristensen L.S., Andersen M.S., Stagsted L.V.W., Ebbesen K.K., Hansen T.B., Kjems J. (2019). The biogenesis, biology and characterization of circular RNAs. Nat. Rev. Genet..

[2] Fischer J.W., Leung A.K.L. (2017). CircRNAs: A regulator of cellular stress. Crit. Rev. Biochem. Mol. Biol..

[3] Cooper D.A., Cortés-López M., Miura P. (2018). Genome-Wide circRNA Profiling from RNA-seq Data. Methods Mol. Biol..

[4] Hsu M.T., Coca-Prados M. (1979). Electron microscopic evidence for the circular form of RNA in the cytoplasm of eukaryotic cells. Nature.

[5] Nigro J.M., Cho K.R., Fearon E.R., Kern S.E., Ruppert J.M., Oliner J.D., Kinzler K.W., Vogelstein B. (1991). Scrambled exons. Cell.

[6] Wu W., Ji P., Zhao F. (2020). CircAtlas: An integrated resource of one million highly accurate circular RNAs from 1070 vertebrate transcriptomes. Genome Biol..

[7] Wang P.L., Bao Y., Yee M.-C., Barrett S.P., Hogan G.J., Olsen M.N., Dinneny J.R., Brown P.O., Salzman J. (2014). Circular RNA is expressed across the eukaryotic tree of life. PLoS ONE.

[8] Szabo L., Morey R., Palpant N.J., Wang P.L., Afari N., Jiang C., Parast M.M., Murry C.E., Laurent L.C., Salzman J. (2015). Statistically based splicing detection reveals neural enrichment and tissue-specific induction of circular RNA during human fetal development. Genome Biol..

[9] Gasparini S., Licursi V., Presutti C., Mannironi C. (2020). The Secret Garden of Neuronal circRNAs. Cells.

[10] De Falco L., Silva N.M., Santos N.C., Huber R.G., Martins I.C. (2021). The Pseudo-Circular Genomes of Flaviviruses: Structures, Mechanisms, and Functions of Circularization. Cells.

[11] Zaiou M. (2020). circRNAs Signature as Potential Diagnostic and Prognostic Biomarker for Diabetes Mellitus and Related Cardiovascular Complications. Cells.

[12] Wang Y., Liu B. (2020). Circular RNA in Diseased Heart. Cells.

[13] Beltrán-García J., Osca-Verdegal R., Nacher-Sendra E., Pallardó F.V., García-Giménez J.L. (2020). Circular RNAs in Sepsis: Biogenesis, Function, and Clinical Significance. Cells.

[14] Nedoluzhko A., Gruzdeva N., Sharko F., Rastorguev S., Zakharova N., Kostyuk G., Ushakov V. (2020). The Biomarker and Therapeutic Potential of Circular Rnas in Schizophrenia. Cells.

[15] de la Peña M., Ceprián R., Cervera A. (2020). A Singular and Widespread Group of Mobile Genetic Elements: RNA Circles with Autocatalytic Ribozymes. Cells.

[16] Wawrzyniak O., Zarębska Ż., Kuczyński K., Gotz-Więckowska A., Rolle K. (2020). Protein-Related Circular RNAs in Human Pathologies. Cells.

[17] Jakobi T., Siede D., Eschenbach J., Heumüller A.W., Busch M., Nietsch R., Meder B., Most P., Dimmeler S., Backs J. (2020). Deep Characterization of Circular RNAs from Human Cardiovascular Cell Models and Cardiac Tissue. Cells.

[18] Conn V.M., Gabryelska M., Marri S., Stringer B.W., Ormsby R.J., Penn T., Poonnoose S., Kichenadasse G., Conn S.J. (2020). SRRM4 Expands the Repertoire of Circular RNAs by Regulating Microexon Inclusion. Cells.

[19] Robic A., Demars J., Kühn C. (2020). In-Depth Analysis Reveals Production of Circular RNAs from Non-Coding Sequences. Cells.

[20] Chen Y.-T., Chang I.Y.-F., Kan C.-H., Liu Y.-H., Kuo Y.-P., Tseng H.-H., Chen H.-C., Liu H., Chang Y.-S., Yu J.-S. (2020). circRNAome Profiling in Oral Carcinoma Unveils a Novel circFLNB that Mediates Tumour Growth-Regulating Transcriptional Response. Cells.

[21] Mo D., Li X., Raabe C.A., Rozhdestvensky T.S., Skryabin B.V., Brosius J. (2020). Circular RNA Encoded Amyloid Beta peptides-A Novel Putative Player in Alzheimer’s Disease. Cells.

[22] Philips A., Nowis K., Stelmaszczuk M., Podkowiński J., Handschuh L., Jackowiak P., Figlerowicz M. (2020). Arabidopsis thaliana cbp80, c2h2, and flk Knockout Mutants Accumulate Increased Amounts of Circular RNAs. Cells.

[23] Philips A., Nowis K., Stelmaszczuk M., Jackowiak P., Podkowiński J., Handschuh L., Figlerowicz M. (2020). Expression Landscape of circRNAs in Arabidopsis thaliana Seedlings and Adult Tissues. Front. Plant Sci..

[24] Dawkins R. (2022). Books Do Furnish a Life.

